# p-ZnO/n-ZnMgO Nanoparticle-Based Heterojunction UV Light-Emitting Diodes

**DOI:** 10.3390/ma15238348

**Published:** 2022-11-24

**Authors:** Islam Mohammad Shafiqul, Toshiyuki Yoshida, Yasuhisa Fujita

**Affiliations:** 1 Interdisciplinary Graduate School of Science and Engineering, Shimane University, 1060 Nishikawatsu, Matsue 690-8504, Shimane, Japan; 2 Interdisciplinary Graduate School of Engineering Sciences, Kyushu University, 6-1, Kasugakouen, Kasuga 816-8580, Fukuoka, Japan; 3 Graduate School of Natural Science and Technology, Shimane University, 1060 Nishikawatsu, Matsue 690-8504, Shimane, Japan

**Keywords:** ZnO, ZnMgO, nanoparticles, heterojunction, light-emitting diodes

## Abstract

Heterojunction light-emitting diodes (LEDs), based on p-type ZnO and n-type ZnMgO nanoparticles, have been demonstrated. ZnMgO nanoparticles were prepared by the thermal diffusion of Mg onto ZnO nanoparticles. p-ZnO/GZO homostructure LEDs and p-ZnO/n-ZnMgO/GZO heterostructure LEDs have been fabricated using ZnO and ZnMgO nanoparticles. By comparing the characteristic results of these diodes, it can be seen that LEDs with the p-ZnO/n-ZnMgO/GZO structure showed better *I–V* characteristics with a lower current density leakage than those with the p-ZnO/GZO LED structure. Moreover, the emission intensity was improved by adding the ZnMgO NP layer to the LEDs. These results show that the ZnMgO NP layer acts as a hetero-barrier layer that suppresses the diffusion of holes into the n-type layer and confines holes to the p-type layer.

## 1. Introduction

Light-emitting diodes (LEDs) have vast potential in the development of solid-state lighting technologies due to their prominent properties. Currently, the fabrication of light-emitting diodes based on ZnO is of great interest because of their advantageous features, such as a wide bandgap of 3.37 eV, excitonic binding energy of 60 meV, and short wavelength UV emission range at room temperature [1,2]. To achieve p-type doping of the ZnO, group V elements are the most promising acceptor candidates [3,4,5,6,7]. Several authors have reported nitrogen-doped p-type ZnO or ZnMgO-based LEDs with single-crystal films using epitaxial growth technologies [8,9,10]. Single-crystal substrates and the epitaxial growth technologies have the problem of high costs. However, the fabrication of nanoparticles (NPs)-based LEDs is easy and inexpensive. 

Recently, ZnO NP has been considered a promising material for the development of semiconductor devices. Some groups have proposed developing nitrogen-doped ZnO NPs using different techniques [11,12,13]. Our group used the DC arc plasma gas evaporation technique to fabricate nitrogen-doped ZnO NPs and followed several conditions to obtain p-type and n-type ZnO NPs [14,15,16,17]. Our group previously reported on the development of semiconductor devices such as TFTs [16] and UV LEDs [18,19] using these ZnO NPs. Fujita et al. [18] reported the synthesis of homojunction UV LEDs using N-doped ZnO NPs on a Ga-doped ZnO (GZO) thin film. Shafiqul et al. [19] reported the world’s first demonstration of an entirely NP-based homojunction UV LED using N-doped p-type ZnO NPs and Ga-doped n-type ZnO NPs. However, the observed performances of these LEDs were not at the expected level. This is because, in a homojunction structure, carriers spread to each layer by diffusion, resulting in a lower carrier density and lower recombination probability.

It was realized that, to overcome this problem, these devices can be constructed based on a heterostructure because carrier confinement is an essential requirement for improving luminescence properties and can lead to high radiative recombination in the active region of the devices. One of the necessary processes of device construction is bandgap engineering by changing the energy structure. The energy structure of the materials has been changed by doping, surface modification, and controlling the size [20,21,22]. Several groups have proposed that ZnMgO alloy is a useful candidate for the hetero-barrier in these devices [23,24,25]. Some authors have discussed heterojunction LEDs using a ZnMgO film layer [26,27,28,29]. Fujita et al. [10] demonstrated ZnMgO:N/ZnO heterojunction UV LEDs by metalorganic vapor phase epitaxy (MOVPE). However, there are no reports of LEDs with heterojunction using ZnO NPs. In this work, a new attempt was made to fabricate ZnMgO-alloy composite NPs by a thermal diffusion process. We constructed heterojunction UV LEDs using N-doped ZnO NPs for the p-type layer and ZnMgO NPs for the n-type layer. The properties of NPs and NP-based LEDs were also investigated. 

## 2. Materials and Methods

A gas evaporation method (ULVAC Inc., Kanagawa, Japan, Model No-GE-970) was applied to synthesize the N-doped ZnO NPs by an arc plasma system and an oxidation system. The N_2_ radicals generated by the arc plasma, together with nitrogen atoms, were incorporated into ZnO NPs during the synthesis process. In this method, we used zinc metal (Nilaco Corporation, Tokyo, Japan, Zn-99.99%) as a Zn source and dry air as an oxygen and nitrogen source. During the synthesis process, the evaporated zinc atoms were reacted with flowing gas, and ZnO NPs were produced. The chamber pressure was regulated at 150 torr and 610 torr through a rotary pump and controlling valve. The arc currents were 30 A and 50 A. The airflow rate inside the chamber was 5 L min^−1^ during the synthesis period. The detailed mechanism of the synthesis of ZnO NPs was described in ta previous report [14,15]. The average particle size of ZnO NPs was between 100 and 200 nm, according to the earlier research work [17,30]. The fabrication of N-doped p-ZnO NPs was carried out at a chamber pressure of 150 torr and an arc current of 30 A [17,19]. 

For the preparation of ZnMgO composite NPs, the prepared n-ZnO NP samples were fabricated at a pressure of 610 torr and an arc current of 50 A, which exhibited n-type conductivity [16]. The n-ZnO NPs (0.1 g) and MgO NPs (UBE Corporation, Tokyo, Japan, MgO-99.98 %, particle size 45~60 nm) (0.05 g) were mixed using a vortex mixer. The ZnO NP and MgO NP mixture was placed in an electric furnace and annealed under an N_2_ gas flow of 0.5 L min^−1^ inside the furnace. The furnace temperature was increased from room temperature to 1000 °C within 6 min and kept constant for 60 min. The NP samples were cooled down to less than 40 °C in 2 h.

**Figure 1:**
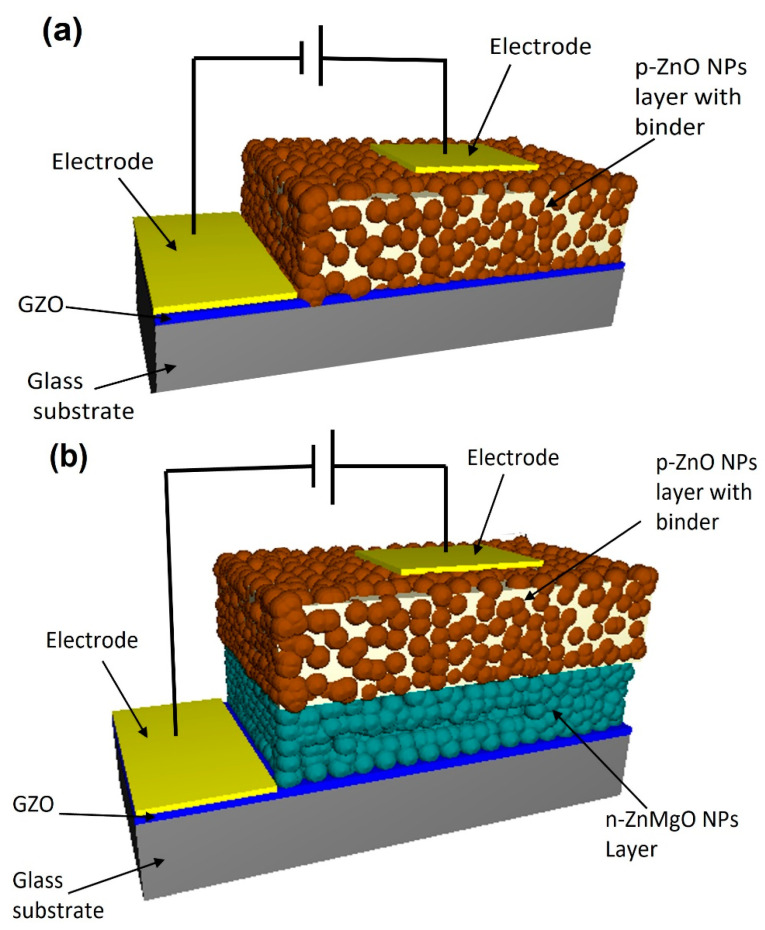
Schematic view of the LEDs based on ZnO NPs. (**a**) the structure of p-ZnO/GZO (**b**) the structure of p-ZnO/n-ZnMgO/GZO.

Two types of ZnO-NP-based LED were designed following the methods earlier [17,19]. One of the LED samples had an N-doped p-ZnO NP layer on the GZO (Ga-doped ZnO) conductive film. Another type of LED had an N-doped p-ZnO NP layer and an n-ZnMgO NP layer on the GZO film as shown in Figure 1a,b, respectively. RF magnetron sputtering (Canon Anelva Corporation, Kanagawa, Japan, Model-400S) was used to prepare the GZO film at 300 °C (5% Ga doped ZnO target) on white glass substrates (thickness of 500 μm and resistivity 4.67 × 10^−4^ Ω cm). The n-ZnMgO-composite NP dispersions were prepared by mixing 10 g pure water with 0.1 g ZnMgO NPs using an ultrasonic homogenizer. Large-size particles were separated from the dispersion by a centrifugal separator (Kubota Corporation, Tokyo, Japan, Model-RA-2724) (3000 G, 1 min). For the formation of the n-ZnMgO NP layer, the separated dispersion was sprayed using an airbrush process onto the GZO electrode film, which was on the hotplate at a temperature of ~300 °C [19].

For the fabrication of the p-type ZnO NP layer in the LEDs, the dispersion was prepared by mixing with N-doped ZnO NPs (0.05 g), isopropyl alcohol (IPA) (0.3) mL, and binder (0.1 g) (Silsesquioxane OX-SQ SI 20; Toagosei Co., Ltd., Tokyo, Japan). The p-ZnO NP dispersions were coated on the GZO electrode films by the spin-coating process. The spin-coating process followed a two-step rotational condition at an initial speed of 1000 rpm for 5 s and was accelerated to a final speed of 4000 rpm for 10 s. The nitrogen-doped ZnO NP-coated layers were sintered by a hotplate at ~300 °C. Gold (Au) electrodes with 30 nm thickness were deposited on both the p-type layer and the GZO film, as a contact electrode, using a vacuum deposition procedure. The thickness of the spin-coated layer was approximately 3 µm and the thickness of the spray-coated layer was around 2 µm according to a previous report [17,19]. 

A diffractometer (Rigaku Smart Lab) with Cuk_α_ radiation was used to measure X-ray diffraction of the NP powder samples. A Horiba FluoroMax-4 spectrofluorometer with an excitation wavelength of 325 nm, using a Xe lamp, was used to detect the photoluminescence (PL) spectra of the ZnO NPs. The current–voltage measurements of the light-emitting diodes were performed using a parameter analyzer (Keysight Technologies B2900A series of High-Resolution SMU module). We evaluated the electroluminescence (EL) spectra of the LEDs from the top side of the p-contact electrode at room temperature using the Ocean Optics QE65000 fiber multi-channel monochrome meter.

## 3. Results and Discussion

**Figure 2:**
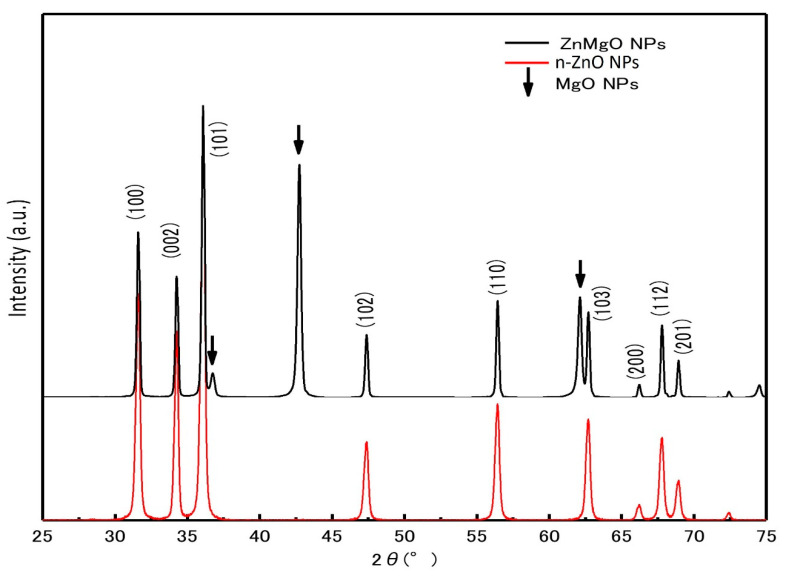
X-ray diffraction patterns of n-ZnO NPs and ZnMgO NPs.

Figure 2 shows the X-ray diffraction (XRD) patterns of the ZnMgO NPs and n-ZnO NP powder samples. The diffraction peaks of n-ZnO NPs and ZnMgO NPs had a hexagonal wurtzite structure, which agrees with ZnO’s standard JCPDS (card no. 36-1451). The additional peaks corresponded to the formation of MgO (No-7-239) [31] compounds from the presence of remaining MgO NPs after annealing. The shift of XRD peaks in ZnMgO NPs occurred toward the lower angle. The lattice constant of ZnMgO NPs was different from the n-ZnO NPs due to the inclusion of the foreign impurity (Mg^+2^) imposed in the host ZnO lattice. This attribution was due to the difference in ionic radius, where the ionic radius of Mg is 0.57 Å, and that of Zn is 0.60 Å [31,32,33]. The lattice constant of the prepared ZnO NPs was 3.259 Å for the a-value and 5.2207 Å for the c-value. On the other hand, the lattice constant of ZnMgO NPs was 3.270 Å for the a-value and 5.2361 Å for the c-value. The ratios of the c/a lattice parameters were 1.6019 for the prepared n-ZnO NPs and 1.6012 for the ZnMgO NPs. The bandgap of the semiconductor was affected by the reduction ratio of the c/a lattice parameters [34]. Vegard’s law expresses a proportional relationship between the lattice constant of the alloy and the concentration of composite elements [35]. For the Zn_1−x_Mg_x_O NP composite, the equation is

*A*_(ZnMgO)_ = *x A*_(Mgo)_ + (1 − *x*) *A*_(ZnO)_ (1)

where A is the lattice constant. From the experimental results, the lattice constant of prepared ZnO NPs is 3.259 Å, and that of ZnMgO is 3.270 Å. The lattice constant that has been reported in the literature for MgO in wurtzite structure is 3.32 Å [36,37]. The Mg content (*x*) in the composite NPs was calculated to be 0.18 (Zn_0.82_Mg_0.18_O).

**Figure 3:**
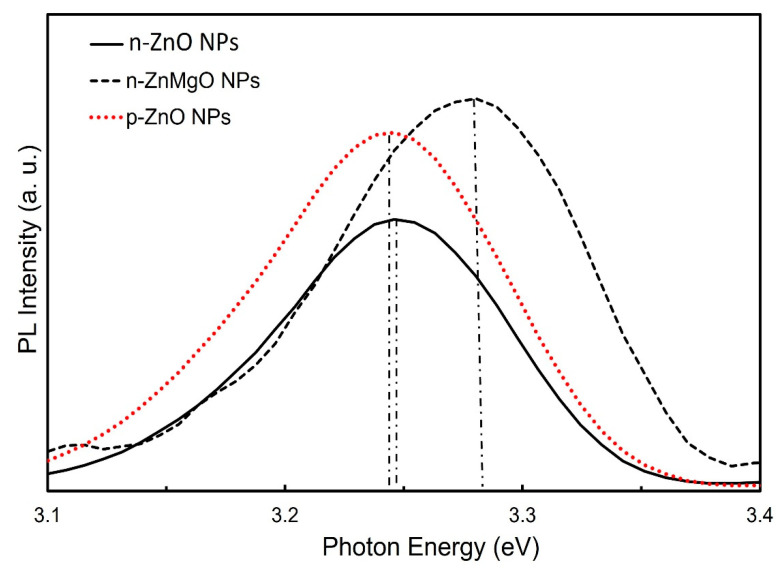
Room-temperature photoluminescence spectra of n-ZnO NPs, n-ZnMgO NPs and p-ZnO NPs.

Figure 3 shows a comparison of the PL spectra results for the n-ZnO NPs (black solid line), n-ZnMgO NPs (black dotted line) and p-ZnO NPs (red dotted line). The PL emission peak of p-ZnO NPs shifted toward the lower-energy side due to the nitrogen acceptor incorporated into ZnO NPs and the appearance its of donor–acceptor pair (DAP) transition [17]. The observed peak position of the band edge luminescence for ZnMgO NPs was at about 3.278 eV and that of n-ZnO NPs was at around 3.246 eV. The emission peak of the PL of the ZnMgO NPs shifted more toward the higher-energy side than the peak energy of the n-ZnO NPs. The difference in PL peak energy between the n-ZnO NPs and ZnMgO NPs was 32 meV. As we know, the bandgap energy of ZnO is 3.37 eV. In this case, assuming that the exciton binding energy does not change with the addition of Mg, the bandgap of ZnMgO NPs can be estimated to be 3.40 eV. The content (*x*) of Mg can be calculated from Equation (2) [35].

*E*_g(ZnMgO)_ = (1 − *x*) *E*_g(ZnO)_ + *x E*_g(MgO)_ (2)

Assuming that the bandgap of MgO is 6.626 eV [38,39], the content (*x*) of Mg in the composite ZnMgO NPs was determined to be 0.01. This is different from the results of the XRD. From the XRD results, the content of Mg (*x*) was 0.18. In this case, the bandgap of ZnMgO was 3.956 eV. The reason for this difference is the lack of uniformity in the thermal diffusion process. The large amount of Mg diffused onto the surface of the ZnO NPs and this distortion was revealed by the XRD. However, the PL emission was less affected because of the many defects on the surface of the NPs. 

**Figure 4:**
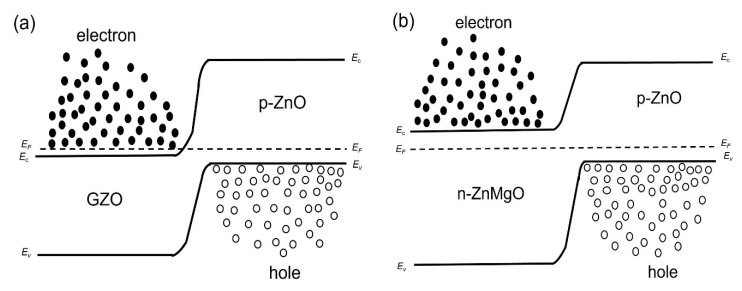
Band diagrams at thermal equilibrium state conditions (band bending is not taken into account) (**a**) p-ZnO/GZO homojunction; (**b**) p-ZnO/n-ZnMgO heterojunction.

**Figure 5:**
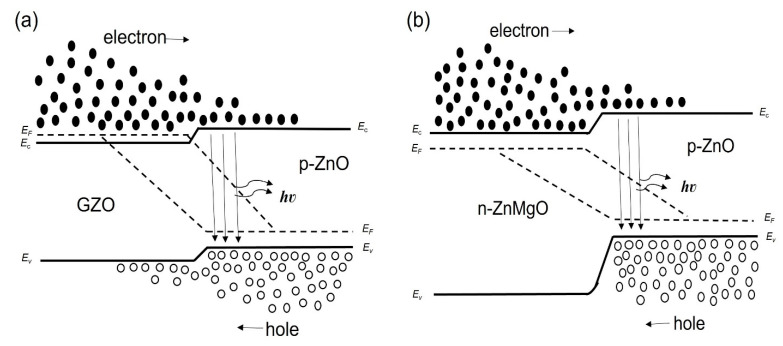
Band diagrams under forward bias conditions (band bending is not taken into account) (**a**) p-ZnO/GZO homojunction; (**b**) p-ZnO/n-ZnMgO heterojunction.

We considered the working mechanism of the ZnO NP-based homo- and heterostructure LEDs. Figure 4a,b show, respectively, the band diagram of the p-ZnO/GZO homojunction and the p-ZnO/n-ZnMgO heterojunction structure at the thermal equilibrium state where E_c,_ E_v,_ and E_F_ are, respectively, the conduction band, valence band and quasi-Fermi level energy. Figure 5a,b show the band diagram of the p-ZnO/GZO homo-structure and the p-ZnO/n-ZnMgO heterostructure, respectively, under forward bias conditions.

**Figure 6:**
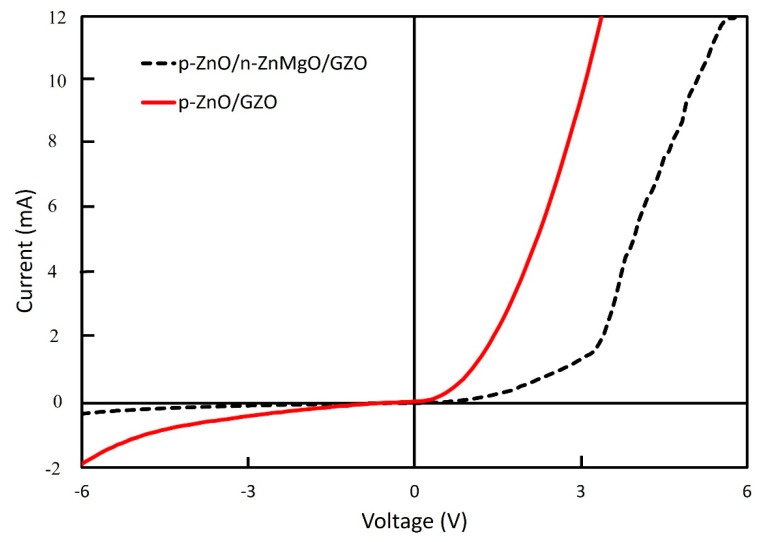
*I–V* characterization of ZnO NP-based LEDs with the structure of p-ZnO/n-ZnMgO/GZO and p-ZnO/GZO.

The *I–V* properties of these LEDs were evaluated. Figure 6 shows the *I–V* characteristics of LEDs at room temperature in a dark environment. Nonlinear rectifying behavior was observed from the *I–V* curve for both structures of LED. The p-ZnO/n-ZnMgO/GZO LED showed superior characteristics compared to the p-ZnO/GZO LED. The turn-on voltage for the LEDs with the structure of p-ZnO/n-ZnMgO/GZO was at around 3.3 V at the forward region, which is in good agreement with the bandgap energy of ZnO. However, this was not observed for the LEDs with the structure of p-ZnO/GZO, which also revealed a higher current density than the LEDs with the structure of p-ZnO/n-ZnMgO/GZO; this is possibly due to the interface difference between the p-ZnO NP and the n-GZO film layers and the interface of the p-ZnO/n-ZnMgO NP layer. On the other hand, the active region for the both structures of LED exists in the p-ZnO NP layer that contains an insulating binder and also the n-type NPs, due to the non-homogeneous particle fabrication process.

**Figure 7:**
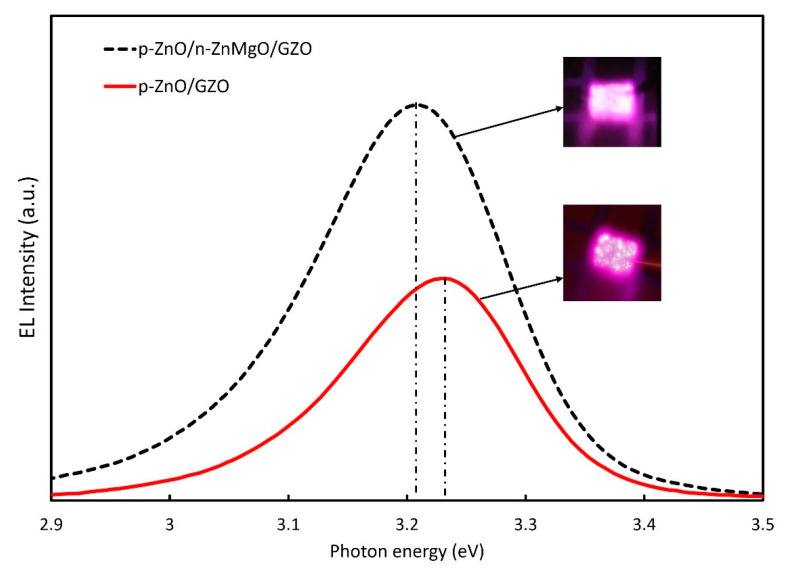
EL comparison for ZnO NP LEDs with the of p-ZnO/n-ZnMgO/GZO and p-ZnO/GZO structures at an injection current of 12 mA and the applied voltages were 5.0 V and 3.0 V, respectively.

The EL spectra of the LEDs with p-ZnO/n-ZnMgO/GZO and p-ZnO/GZO structures at room temperature are presented in Figure 7. By inserting the ZnMgO NP energy-barrier layer, the intensity of the EL spectra increased compared to that without the ZnMgO NP layer. In the p-ZnO/GZO structure, holes are injected into the n-type GZO layer. However, the holes injected into the GZO layer do not contribute to luminescence because GZO has a high electron concentration and does not emit light. The p-ZnO layer has a lower hole concentration than the electron concentration in the GZO. The LED with p-ZnO/GZO structure shows a charge imbalance, which means that more electrons are congregated in the p-ZnO layer with holes. The configuration with an inserted ZnMgO layer (p-ZnO/n-ZnMgO/GZO) is a structure that balances the concentration of electrons and holes. The reason for this was that the energy barrier layer blocked the injection of the holes into the n-type layer and increased the concentration of holes in the active region, as shown in Figure 5. As a result, more holes contributed to luminescence in the LED with p-ZnO/n-ZnMgO/GZO structure and enhanced the emission compared to the LED with p-ZnO/GZO structure.

The peak energy of EL emission for p-ZnO/GZO LEDs was found to be around 3.23 eV. On the other hand, the peak energy of EL spectra for the LED with an inserted ZnMgO NP layer (p-ZnO/n-ZnMgO/GZO) was observed at about 3.21 eV. When comparing the EL spectra for both LEDs, the emission energy peaks were shifted toward the lower-energy side. The results prove that the EL emission occurred by carrier recombination in the p-ZnO NP layer. The lower direction of the peak energy shift is due to the decreased bandgap energy, caused by the heating effect of the rising temperature [40], and the increase in carrier density. In this case, for the structure of p-ZnO/GZO LEDs, the carrier will concentrate in the p-ZnO NP layer with an insulating binder, resulting in a higher excitation density and redshift. The holes from the p-ZnO NP layer can flow to the GZO layer, so the carrier density should be lower than that of LED with the ZnMgO NP layer. On the other hand, the structure of p-ZnO/n-ZnMgO/GZO LEDs contained a ZnMgO NP layer, resulting in a temperature increase, due to the resistivity of the ZnMgO layer, and the large redshift occurred. The temperature of the LEDs with the structure of p-ZnO/n-ZnMgO/GZO and p-ZnO/GZO was calculated using the Varshni model [40], Equation (3), in this case assuming no redshift due to increased excitation carrier density.

*E*_g_ (*T*) = *E*_g_ (*T* = 0) − α*T*^2^/ (*T* + *β*) (3)

where *E*_g_ (*T* = 0) = 3.44 eV for ZnO at 0 K [41]. The temperature coefficients are *α* = −5.5 × 10^−4^ eV K^−1^ and *β* = −900 K for temperatures up to 300 K [42,43]. The temperature calculated from the peak energy (3.21 eV) of EL spectra for the LEDs with the structure of p-ZnO/n-ZnMgO/GZO was approximately 440 K. The thermal energy of the LEDs with the structure p-ZnO/n-ZnMgO/GZO at 440 K is around 38 meV, and is larger than that of the heterobarrier. The temperature with the structure of p-ZnO/GZO was approximately 426 K. 

As mentioned above, the Mg content of the ZnMgO NPs influenced the ZnO structure due to bandgap tuning. Considering these results, the authors conclude that the ZnMgO NP layer acted as an n-type layer in the diode and created a hetero-barrier for hole injection in the active region of the LEDs.

## 4. Conclusions

By compositing Mg into ZnO NPs, the bandgap engineering of ZnMgO NPs was confirmed and the characterization was revealed by the results, including XRD and PL. The ZnMgO NP layer was inserted into the LEDs as an energy barrier layer, which created carrier confinement in the active region of the devices and carried out electron–hole balance radiative recombination. The diode characteristics of the LEDs with the structure of p-ZnO/n-ZnMgO/GZO were significantly improved, as well as the EL emission, which was comparable to that of the p-ZnO/GZO LED structure. In light of these results, the structure and fabrication processes of heterostructure LEDs with Zn_1−x_Mg_x_O could be optimized for significant progress in optoelectronic devices.

## Data Availability

The data that support the findings of this study are available on request from the corresponding author. The data are not publicly available due to privacy or ethical restrictions.
